# The association between *HSD3B7* gene variant and Parkinson's disease in ethnic Chinese

**DOI:** 10.1002/brb3.913

**Published:** 2018-02-17

**Authors:** Zhong‐Jiao Lu, Ling Wang, Xiao‐Yi Sun, Jun‐Ying Li, Lan Cheng, Nan‐Nan Li, Rong Peng

**Affiliations:** ^1^ Department of Neurology West China Hospital Sichuan University Chengdu Sichuan Province China

**Keywords:** bile acid synthesis, gene polymorphism, *HSD3B7*, neuroprotection, Parkinson's disease

## Abstract

**Objectives:**

Studies at the genomewide level of Parkinson's disease (PD) suggested a significant association between the Hydroxy‐delta‐5‐steroid dehydrogenase, 3 beta‐ and steroid delta isomerase 7 (*HSD3B7*) gene rs9938550 variant and a decreased risk for PD. But its effect has only been discussed in Caucasian populations, and no phenotypic characteristics were included. To investigate the novel variant for PD in Chinese Han populations, we performed an association analysis of rs9938550 variant in a large cohort.

**Methods:**

Using a case–control methodology, a total of 2,239 subjects (1,072 sporadic patients with PD and 1,167 control) were genotyped and the genetic association was analyzed.

**Results:**

No significant association was found between allele A of rs9938550 and PD in the entire cohort (*p *=* *.079). However, the frequency of allele A was lower in late‐onset PD (LOPD) as compared with controls older than 50 years (OR = 0.62, 95% CI: 0.45–0.85, *p*_adjust_ = .002). Relatively lower Unified Parkinson's Disease Rating Scale scores were demonstrated in mid‐ to late‐stage PD with GA + AA genotypes than GG genotype (*p*_adjust_ = .018), while other clinical features were similar between carriers and noncarriers.

**Conclusions:**

Our results support that the *HSD3B7* rs9938550 variant, which is likely linked to bile acid biosynthesis, reduces the risk of LOPD in Chinese patients and might induce a benign clinical performance.

## 1 INTRODUCTION

Parkinson's disease (PD) is the most common movement disorder typically characterized by motor disability (Tysnes & Storstein, 2017). It has an age‐dependent prevalence, and its burden at the population level is estimated to expand dramatically as the size of elderly population grows (Dorsey et al., 2007; Trinh & Farrer, 2013). The majority of patients with PD are sporadic forms, probably resulting from the interactions between genetic and environmental factors (Migliore & Coppede, 2009). Despiting several disease‐modifying drugs have been developed, there is currently no cure for PD. Therefore, identifying genetic susceptibility for PD will be beneficial for detecting the pathogenesis and tailoring therapy.

Genomewide association study (GWAS), an effective approach for understanding the genetic basis of complex diseases, has recently identified several novel candidate loci for PD (Hill‐Burns et al., 2014; Tan, Jiang, Tan, & Yu, 2014). Rs9938550, a single‐nucleotide polymorphism (SNP) in the 3β‐hydroxy‐Δ5‐C27‐steroid dehydrogenase/isomerase (*HSD3B7*) gene (16p 11.2), may play a vital role in an unknown pathogenesis related to the process of bile acid and steroid metabolism (Nalls et al., 2014). When combining the finding from a pathway‐based association study, there is a potential contribution of rs9938550 to *HSD3B7* on PD (Song & Lee, 2013). In the classical pathway, *HSD3B7* catalyzes the second step of bile acid formation (Monte, Marin, Antelo, & Vazquez‐Tato, 2009), and its mutations may reduce the synthetic capability (Cheng et al., 2003). Interestingly, when the main product, chenodeoxycholic acid (CDCA), converts into a β‐configuration ursodeoxycholic acid (UDCA) and its taurine‐conjugated form (TUDCA), the anti‐apoptotic effect is demonstrated (Ackerman & Gerhard, 2016; Amaral, Viana, Ramalho, Steer, & Rodrigues, 2009; Hirano, Masuda, & Oda, 1981). In a PD module, UDCA significantly attenuated programed cell death events and protected human dopaminergic SH‐SY5Y cells through the PI3K‐Akt/PKB signaling pathways, similarly, UDCA deregulated the level of rotenone‐induced apoptosis by modulating mitochondrial dysfunction (Abdelkader, Safar, & Salem, 2016; Chun & Low, 2012). TUDCA was also found to activate the prosurvival Akt pathway, diminishing the neurodegeneration in a vivo module of PD (Castro‐Caldas et al., 2012).

However, current work to discover the physiological roles of bile acids in neurodegenerative conditions mostly focused on UDCA and TUDCA, little has been performed on the initial stage that *HSD3B7* gene is involved (Ackerman & Gerhard, 2016). Given the limited power to identify disease gene by GWAS or pathway‐based approaches and the neuroprotective potential of bile acids for PD, replication studies are needed from different ethnic groups (Wang, Li, & Hakonarson, 2010). We aimed to investigate the relationship between SNP rs9938550 in *HSD3B7* gene and PD using SNP array on a large sporadic PD cohort of Chinese samples.

## 2 MATERIALS AND METHODS

### 2.1 Subjects

A total of 1,072 sporadic PD cases were recruited for this study (589 males, 483 females, mean age at onset, AAO 52.19 ± 10.59) at the Department of Neurology of West China Hospital, Sichuan University. All cases underwent a neurological evaluation that employed PD diagnostic criteria based broadly on the United Kingdom PD Society Brain Bank (UKPDBB) Criteria (Hughes, Daniel, Kilford, & Lees, 1992) by movement disorder specialists, while those with a positive family history of PD were excluded. According to a cluster analysis, patients were subcategorized into early‐onset (AAO < 50, EOPD) and late‐onset (AAO ≥ 50, LOPD) groups (Post, Speelman, & de Haan, 2008; Ross et al., 2008). Unified Parkinson's Disease Rating Scale (UPDRS) scores and Hoehn–Yahr (HY) stage in the OFF state were recorded to demonstrate the clinical stages (Goetz et al., 2004; Post, Merkus, de Bie, de Haan, & Speelman, 2005). Control subjects (613 males, 554 females, mean age 51.96 ± 15.41) were ethnic‐, age‐, and gender‐matched, and had no evidence of any neurological disorder. A written informed consent was obtained from each subject involved in the study and ethical approval was provided by the Ethics Committee of Sichuan University.

### 2.2 Genetic analysis

DNA extraction and SNP genotyping were performed using standard protocols as described previously (Liao et al., 2014). Briefly, polymerase chain reaction (PCR) and detection primers were designed by the MassArray Assay Design 3.0 software (Sequenom). About 15 ng of genomic DNA was amplified by primers flanking the targeted sequence, followed by dephosphorylation and allele‐specific primer extension. Products were loaded into a Spectro‐Chip and subjected to a matrix‐assisted laser desorption/ionization time‐of‐flight (MALDI‐TOF) mass spectrometry. The Sequenom MassArray Typer software (Sequenom) conducted the data analysis. Thus, we detected a missense mutation (c.748A>G) in the *HSD3B7* gene, substituting threonine for alanine at residue 250 (p.Thr250Ala).

### 2.3 Statistical analysis

Statistical analysis was performed using SPSS software version 18.0 (SPSS Inc., Chicago, IL, USA) and SHEsisPlus (Shi & He, 2005). Fisher's exact test was used to check Hardy–Weinberg equilibrium (HWE) of each SNP for subjects. Continuous variables were evaluated by a *t* test or a Mann–Whitney *U* test according to distribution (normal or skewed). Categorical variables were compared using a chi‐squared test to analyze the distribution of genotype and allele. SNP associations were evaluated using logistic regression models, which were further performed after adjustment for age, gender, and other risk factors through odds ratios (OR) with 95% confidence intervals (CI). A two‐tailed *p*‐value < .05 was considered significant.

## 3 RESULTS

A total of 2,239 subjects (1,167 controls and 1,072 cases) were included in the analysis. There was no evidence of deviation from HWE in SNP rs9938550 in the population (*p *=* *.319). No differences in age or gender between cases and controls were found (*p *=* *.853; *p *=* *.270, Table S1). The minor allele frequency of rs9938550 in patients with PD is 6.2%, and it did not differ significantly when compared to controls of 7.4% (*p *=* *.079, OR = 0.81, 95% CI: 0.64–1.03). No difference was observed yet in the frequencies of genotypes between two groups in overall PD population (*p *=* *.314).

Multivariate regression analysis with adjustment for age, gender indicated that the distribution of allele A was not significantly different between the controls and PD groups in both genders, at all ages, also no association was found between EOPD and younger controls (<50 years) (Table 1). However, the allele A was significantly less frequent in the LOPD subgroup than in controls (≥50 years) when combining both genders (*p *=* *.002, OR = 0.62, 95% CI: 0.45–0.85) or considering each gender alone (*p *=* *.013, OR = 0.60, 95% CI: 0.40–0.9, for males; *p *=* *.049, OR = 0.62, 95% CI: 0.39–0.99, for females). In an exploratory analysis, PD samples were divided into two subgroups (GA + AA carriers and GG carriers) to compare clinical characteristics (Table 2). Based on the HY stage, mid‐ to late‐stage (stage III to V) (Coelho & Ferreira, 2012) patients with genotypes GA + AA had the evidently lower UPDRS scores than GG carriers after adjusting for age, gender, and disease duration (*p *=* *.018). But there was no correlation in the clinical presentation for AAO, gender, onset symptoms, or HY stage between two subgroups (Table 2).

**Table 1:** Allelic distribution of HSD3B7 gene in Chinese patients with PD and at different ages at onset

	All ages			<50 years			≥50 years		
	HC	PD	*p* value	HC	EOPD	*p* value	HC	LOPD	*p* value
Total^a^									
A, *N* (%)	172 (7.4)	133 (6.2)	.079	60 (5.8)	62 (7.3)	.279	112 (8.7)	71 (5.4)	.002^b^
G, *N* (%)	2,162 (92.6)	2,011 (93.8)		982 (94.2)	780 (92.7)		1,180 (91.3)	1,231 (94.6)	
Females									
A, *N* (%)	77 (7.0)	61 (6.3)	.596	26 (5.2)	31 (7.5)	.168	51 (8.5)	30 (5.3)	.049^b^
G, *N* (%)	1,031 (93.0)	905 (93.4)		478 (94.8)	379 (92.5)		553 (91.5)	526 (94.7)	
Males									
A, *N* (%)	95 (7.8)	72 (6.1)	.149	34 (6.4)	31 (7.1)	.607	61 (8.9)	41 (5.4)	.013^b^
G, *N* (%)	1,131 (92.2)	1,106 (93.9)		504 (93.6)	401 (92.9)		627 (91.1)	705 (94.6)	

PD, Parkinson's disease; HC, healthy control; EOPD, early‐onset PD; LOPD, late‐onset PD.

a Test by logistic regression after adjusting for age, gender.

b *p *<* *.05; patients with PD were subcategorized by age at onset.

**Table 2:** Clinical characteristics of patients with PD between A allele carriers and noncarriers

	GA + AA	GG	*p* value
Age at onset, mean (*SD*)			
Total cohort	50.54 (10.5)	52.41 (10.6)	.061
EOPD^a^	41.47 (5.7)	41.69 (5.9)	.713
LOPD^a^	58.41 (6.6)	59.08 (6.7)	.390
Gender, *N* (%)			
Male	68 (53.5)	521 (55.1)	.776
Female	59 (46.5)	424 (44.9)	
Onset symptoms, *N* (%)			
Resting tremor	70 (55.1)	452 (47.8)	.128
Bradykinesia	43 (33.9)	298 (31.5)	
Rigidity	7 (5.5)	82 (8.7)	
Mixed symptoms	7 (5.5)	106 (11.2)	
Others^b^	0 (0.0)	7 (0.7)	
HY, *N* (%)			
1–2.5	100 (78.7)	694 (73.4)	.235
3–5	27 (21.3)	251 (26.6)	
UPDRS, mean (*SD*)^c^			
HY: 1–2.5	38.10 (17.7)	38.40 (17.2)	.870
HY: 3–5	63.70 (16.9)	74.77 (23.2)	.004^d^

EOPD, early‐onset PD; LOPD, late‐onset PD; UPDRS, Unified Parkinson's Disease Rating Scale; HY, Hoehn–Yahr stage.

a Mann–Whitney *U* test adopted.

b Including pain, weakness, symptoms of autonomic dysfunction, and so on.

c *T* test adopted.

d *p *<* *.05.

## 4 DISCUSSION

In Chinese Han populations, especially for the older cohorts, Allele A of rs9938550 is likely associated with a reduced risk of developing PD, which is supported by the prior works in old Caucasians (Nalls et al., 2014; Song & Lee, 2013). But we could not replicate this result in our EOPD subgroup. Given that advancing age is an important risk factor for PD, as its onset and prevalence increase particularly after age 50 (Elbaz et al., 2002; Pezzoli et al., 2014). These findings suggested that SNP rs9938550 in *HSB3D7* gene presents an effect in the main population of patients with PD. To further detect the relationship between the variant and PD, we added our results into a meta‐analysis based on the PDGene database (http://www.pdgene.org) (Nalls et al., 2014). No statistical heterogeneity was found among 12 included studies undergoing the same UKPDBB criteria (*I* ^2^ = 31). Notably, the result also revealed that allele A was less frequent in PD than in controls (*p *=* *5.81 × 10^−6^, OR = 0.90, 95% CI: 0.88–0.94, Figure S1), suggesting *HSD3B7*, in a protective manner, is a potential candidate locus for PD (Ackerman & Gerhard, 2016).

Moreover, older AAO was associated with a more severe PD phenotype, we focused on the relationship between allele A and patients of a mid‐ to late‐stage (Pagano, Ferrara, Brooks, & Pavese, 2016). Consistent with our hypothesis, those patients with GA + AA genotypes had lower UPDRS scores compared to GG genotype carriers, suggesting that rs9938550 variant tended to feature a favorable performance in the advanced stage of PD. However, it failed to reach statistical significance in other clinical features, which was likely a result of a relatively small sample size in GA + AA subgroup and an age‐related effect.

Considering the neuroprotective effects of bile acids, a set of neurodegenerative diseases have been reported, including PD, Alzheimer's disease (AD), and Huntington's disease (HD), but the available data for *HSD3B7* are limited (Ackerman & Gerhard, 2016; Ramalho et al., 2013; Rodrigues et al., 2000). Astrocyte expressing *HSD3B7* is responsible for degradation of oxysterol, the active oxidized product of cholesterol, which can be used as a marker of brain atrophy in patients holding aging neurons with AD and HD (Leoni & Caccia, 2011; Rutkowska, Preuss, Gessier, Sailer, & Dev, 2015). Additionally, the production of the dafachronic acids, a nuclear receptor for bile acids, can be regulated by a conserved 3 beta‐*HSD* to participate in cholesterol, bile acid metabolism, and longevity (Wollam et al., 2012). It might be conceivable that an age‐specific effect of allele A in *HSD3B7* locus can modulate the neuroprotection to patients with PD.

Our study, although exploratory, has some limitations. Potential gene–gene and gene–environment interactions were not taken into account. Concerning the complex genetic architecture of *HSD3B7* locus and lack of sufficient power of statistical methods used in individual SNP assays, the genetic variants identified to be associated with PD usually explain a relatively small proportion of the heritability. Currently, only a few studies demonstrated that primary bile acid biosynthesis contributed to neuron degeneration when employing pathway‐based GWAS to exploit the collective effects of a number of causal variants and to improve the power of detection (Huang, Martin, Vance, & Cai, 2014). Then, the sample size, albeit relatively large, is somewhat limited when considering the low frequency of allele A in our population and ethnic heterogeneity.

Taken together, we observe a trend toward significance with an *HSD3B7* variant rs9938550 to PD in a Chinese Han population while A allele of it is associated with a decreased risk of LOPD. Our data also suggest that patients with A allele at mid‐ to late‐stage appear to have benign clinical presentations. Nevertheless, the assessment of this variant is insufficient and the variability in biomarker in patients with different AAO should be considered, therefore, follow‐up work is currently underway to determine potentially protective effects of allele A and their causal mechanisms by more replication studies from other regions.

## CONFLICT OF INTEREST

None.
