# Supplementary figures and images for: The association between HSD3B7 gene variant and Parkinson's disease in ethnic Chinese
Source: Brain Behav. 2018 Feb 17;8(4):e00913. doi: 10.1002/brb3.913 (PMC5893344; doi:10.1002/brb3.913)

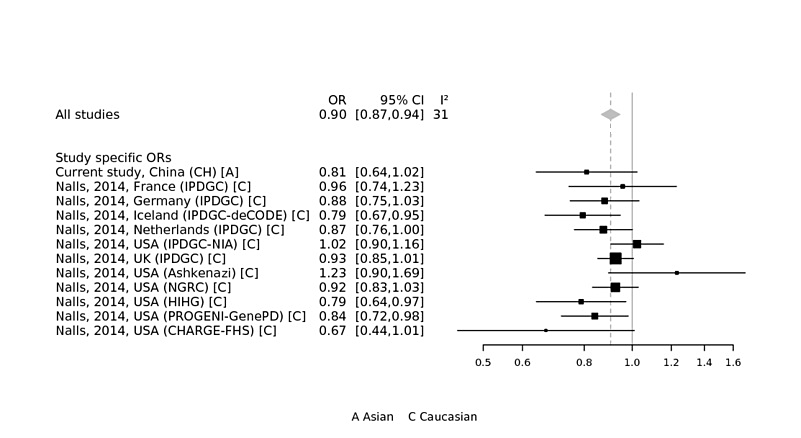

Supplement: Supplementary file 1 [file BRB3-8-e00913-s001.tif]
