# Supplementary material for: The association between HSD3B7 gene variant and Parkinson's disease in ethnic Chinese
Source: Brain Behav. 2018 Feb 17;8(4):e00913. doi: 10.1002/brb3.913 (PMC5893344; doi:10.1002/brb3.913)
Supplement: Supplementary file 2 [file BRB3-8-e00913-s002.doc]

**Table S1** Characteristics of all PD patients and controls

|  | Patients (*N*=1072) | Controls (*N*=1167) | *P* value |
| --- | --- | --- | --- |
| Age, mean (SD) | 52.19(10.59)^a^ | 51.96(15.41) | 0.853^b^ |
| Gender, N (%) |  |  | 0.270^c^ |
| Male | 589(54.9) | 613(52.5) |  |
| Female | 483(45.1) | 554(47.5) |  |
| Disease duration, median(IQR) | 3(6.0) | NA |  |
| Genotype distribution, N(%) |  |  | 0.314^c^ |
| GG | 945(88.2) | 1004(86) |  |
| GA | 121(11.3) | 154(13.2) |  |
| AA | 6(0.6) | 9(0.8) |  |

PD: Parkinson’s Disease; ^a^ Age at onset for patients; ^b^ T test adopted; ^c^ χ2 test adopted
